# Interaction between Neuroanatomical and Psychological Changes after Mindfulness-Based Training

**DOI:** 10.1371/journal.pone.0108359

**Published:** 2014-10-20

**Authors:** Emiliano Santarnecchi, Sicilia D’Arista, Eutizio Egiziano, Concetta Gardi, Roberta Petrosino, Giampaolo Vatti, Mario Reda, Alessandro Rossi

**Affiliations:** 1 Department of Medicine, Surgery and Neuroscience, University of Siena, Siena, Italy; 2 Department of Neurological, Neurosurgical and Behavioral Sciences, University of Siena, Siena, Italy; 3 Berenson-Allen Center for Non-Invasive Brain Stimulation, Beth Israel Deaconess Medical Center, Harvard Medical School, Boston, Massachusetts, United States of America; 4 Department of Molecular and Developmental Medicine, University of Siena, Siena, Italy; King's College London, United Kingdom

## Abstract

Several cross-sectional studies have documented neuroanatomical changes in individuals with a long history of meditation, while a few evidences are available about the interaction between neuroanatomical and psychological changes even during brief exposure to meditation. Here we analyzed several morphometric indexes at both cortical and subcortical brain level, as well as multiple psychological dimensions, before and after a brief -8 weeks- Mindfulness Based Stress Reduction (MBSR) training program, in a group of 23 meditation naïve-subjects compared to age-gender matched subjects. We found a significant cortical thickness increase in the right insula and the somatosensory cortex of MBSR trainees, coupled with a significant reduction of several psychological indices related to worry, state anxiety, depression and alexithymia. Most importantly, an interesting correlation between the increase in right insula thickness and the decrease in alexithymia levels during the MBSR training were observed. Moreover, a multivariate pattern classification approach allowed to identify a cluster of regions more responsive to MBSR training across subjects. Taken together, these findings documented the significant impact of a brief MBSR training on brain structures, as well as stressing the idea of MBSR as a valuable tool for alexithymia modulation, also originally providing a plausible neurobiological evidence of a major role of right insula into mediating the observed psychological changes.

## Introduction

Mindfulness Based Stress Reduction (MBSR) is a mindfulness meditation-based program in which participants are invited to connect with their physical sensations, perceptions, emotions, cognitions and behaviour, over a period of 8 weeks, with a “non-judgmental” attitude [1], [2]. In general, mindfulness meditation based programs lead to changes in the attitude of the practitioners towards their thoughts, sensations and emotions. This can be considered useful in reducing stress symptoms in non-clinical populations [3], [4] and may help patients cope with a wide variety of clinical conditions, such as anxiety [5], [6], depression [7], [8], substance abuse [9] and chronic pain [10].

A growing body of literature has proposed possible neuroanatomical changes due to mindfulness and other meditation style trainings, mainly using a cross-sectional approach. For instance, almost ten years ago Lazar and colleagues [11] have originally reported a significant increase of cortical thickness in the right insula and frontal lobes of expert meditators (insight meditation), by the means of surface-based cortical thickness measurement. Afterwards, other studies using voxel based morphometry (VBM) have suggested an increase of grey matter (volume or concentration) in different brain regions, following long-term meditation: Hölzel and colleagues documented changes in the right hippocampus, the insula and the left temporal lobe after “Insight Meditation” training [12]; Vestergaard-Poulsen et al. found changes in left superior and inferior frontal gyrus, cerebellum and left fusiform gyrus in Tibetan Buddhist meditators [13]; Luders et al. found changes in right orbito-frontal cortex, right thalamus, left inferior temporal lobe and right hippocampus in Zen, Vipassana or Samatha practitioners [14]; Grant et al. found differences in right dorsal anterior cingulate cortex and secondary somatosensory cortex in Zen meditators [15]. Due to the cross-sectional nature of these studies, no causal relationship between meditation practice and brain anatomical changes can be drawn. Moreover, it is worthwhile to note that all these studies were conducted on expert meditators belonging to different traditions. These may include focused attention meditation, which involves moment by moment selective attention focused on a particular object (e.g., sensations associated with the breath or body), or open monitoring meditation, which involves an open awareness of any stimuli that occur in the present moment, but there are also meditation practices that use mantras or visualizations. It is evidently impossible to generalize the effect of various meditative methods on brain structure and function and it is not the purpose of this paper to compare different meditative methods.

A recent study by Hölzel et al. documented the effect of an 8-week MBSR training in meditation naïve subjects without clinical symptoms [16]. Using VBM, the authors demonstrated anatomical changes after the MBSR program, showing an increase in grey matter concentration values of posterior cingulate cortex, temporal-parietal junction and cerebellum in the MBSR group in respect to a wait-control group. Considering the different available approaches for the neuroanatomical changes evaluation - which are often exclusively focused on one specific brain property (e.g., cortical volume, concentration, thickness) - complementary investigations are needed in order to confirm and expand these findings. Moreover, whether such morphometric changes also correspond to modifications at behavioral and psychological levels remains to be investigated, opening the interesting opportunity to understand the neurobiological underpinnings of the documented effects of MBSR on depression and anxiety symptomatology. In the present study, we aimed to expand previous findings by adopting multiple methods for longitudinal gray matter morphometric analyses. Specifically, the impact of an 8-week MBSR training program on brain cortical and subcortical structures in meditation-naïve subjects has been evaluated by the estimation of grey matter volume (through optimized voxel-based morphometry – VBM) [17], [18] and through grey matter thickness evaluations (voxel-based cortical thickness – VBCT) [19]. VBCT has been used as a complementary analysis to VBM, since it offers increased sensitivity to morphometric properties of more convoluted brain regions and consequently guarantees an increased probability of detecting modifications in deep brain structures which might be involved in meditation practice [20]. Furthermore, we additionally correlated longitudinal changes in anatomical structures with several psychological indices evaluated before and after the MBSR training.

## Experimental Procedures

### 2.1 Participants

Forty-eight right-handed participants were recruited from the responders to an announcement for an 8-week mindfulness-based training, promoted by the Department of Neurological, Neurosurgical and Behavioral Sciences, University of Siena, Italy. All participants declared to be naïve to mindfulness and meditation practice. Further inclusion criteria were a score >27 on the Mini-Mental Status Examination (MMSE), no history of psychiatric or neurological disorders, and suitability for participation in a magnetic resonance imaging (MRI) study. Participants were randomly assigned to 2 groups. The MBSR group consisted of 24 subjects (age 31±4 years, 13F-11M) who underwent a psychological and neuroradiological evaluation performed before and after the MBSR training, while the 24 remaining subjects (age 30±4 years, 12F–12M) underwent the same pre-post multidisciplinary assessment without attending the training, at an equivalent time interval of 8 weeks. The adopted wait-list solution was preferred to a “no-contact group”, in order to control for potential bias due to group- differences in terms of motivation. All study protocols and consenting procedures were approved by the Ethical Committee of the University of Siena, written informed consent was obtained from all participants according to the Declaration of Helsinki.

### 2.2 MBSR program

MBSR is an 8-week intensive program that involves daily exercises in focusing attention on the present moment, as described by Kabat-Zinn and colleagues [2]. Core components include practicing body scanning, sitting meditation, walking meditation and mindful stretching movements. The program features weekly 2.5 hour long in-class sessions; on each of the six days between classroom sessions, participants are asked to practice the meditation based exercises on their own for at least 45 min each day [21]. Between class 6 and 7 the participants are invited to a whole day silent retreat (7 hours). The program was conducted by 2 instructors (S.D., C.G.) that regularly teach MBSR. The participants were asked to write about their daily meditation activities describing the kind of meditation performed and the length of the practice.

The meditation training, by which individuals develop the ability to direct and maintain an open and “non-judgmental” attention towards the present moment, is based on learning to use different anchoring tools to remain focused on the present: the bodily perception linked to breathing, spontaneous sensations in the body and sensations linked to the position of the body in space, internal and external sounds, taste, thoughts and emotions (here described as “thoughts with a bodily sensation”). The whole program has an attitudinal framework exploring different facets of what is described as “non-judgmental” attention, with the capacity to observe the anchoring object before naturally following interlocked thoughts. The program additionally consists of an informal practice designed to allow the subject to direct awareness towards specific observations of everyday life, such as the moment of eating, capturing pleasant and unpleasant events, dialogical interactions, and more.

### 2.3 Neuroradiological Acquisition

MRI examinations of all participants were performed at a 1.5 Tesla Philips Intera Scanner (Philips Medical Systems, Best, The Netherlands). T1-weighted Fast Field Echo (FFE) 1-mm thick images of the entire brain (TE = 4.6 ms, TR = 30.00 ms, flip angle = 30.00, FOV = 250 mm, matrix 256×256, slices = 150), were acquired in the axial plane parallel to the anterior and posterior commissure. To verify the absence of grey and white matter lesions or hyperintensities, a thorough neuroradiological examination also included (i) 1-mm coronal FFE, (ii) 3-mm T2-weighted Turbo Fluid Attenuated Inversion Recovery (FLAIR) and (iii) 3-mm T2-weighted FLAIR images.

### 2.4 Psychological Evaluation

Participants completed the following psychological tests under the supervision of three expert psychologists and psychiatrists (M.R., E.E., R.P.), before and after the MBSR training course.

#### 2.4.1 Toronto Alexithymia Scale (TAS-20)

The TAS-20 is a self-report questionnaire that reveals the alexithymia construct. Alexithymia refers to a condition in which people have trouble identifying and describing emotions and tend to minimize their emotional experience and focus their attention externally. In the initial validation study [22], exploratory factor analysis of the TAS-20 yielded a three factor structure congruent with the theoretical construct of alexithymia: 1) difficulty in identifying feelings and bodily sensations of emotional arousal; 2) difficulty in describing feelings to others, 3) externally-oriented thinking. It is composed of 20 items rated on a 5-point Likert scale, ranging from 1 (strongly disagree) to 5 (strongly agree). Total scores range from 20 to 100. The validity of the three factor structure has been demonstrated in the Italian version by confirmatory factor analysis [23].

#### 2.4.2 Penn State Worry Questionnaire (PSWQ) [24]

This is a self-report questionnaire designed to measure some of the important features of clinically relevant worrying, namely the (1) generality of worrying over time and situations, the (2) intensity/excessiveness of worrying, and the (3) uncontrollability of worrying. PSWQ is made up of a list of 16 worry-based dysfunctional characteristics. A participant has to indicate how typical these characteristics are for him/her on a five-point Likert scale from 1 (Not at all typical of me) to 5 (Very typical of me). Higher scores indicate greater worry.

#### 2.4.3 State-Trait Anxiety Inventory (STAI) - Form Y [25]

This is a self-report questionnaire for measuring anxiety in adults. It clearly differentiates between the temporary condition of “state anxiety” and the more general and long-standing quality of “trait anxiety”. The STAI is composed of a total of 40 self-report items referring to State anxiety as the “intensity of the anxiety emotion in this moment”, and trait anxiety as the more stable general personal disposition.

#### 2.4.4 Beck Depression Inventory II (BDI II) [26]

This measure consists of 21 items multiple-choice self-report items to assess the intensity of depression in clinical and normal populations. BDI-II values different aspects of depression: symptoms of depression such as hopelessness and irritability, cognition such as guilt, as well as physical symptoms such as fatigue, weight loss and lack of interest in sex. The measures ask respondents to endorse statements characterizing how they have been feeling throughout the previous 2 weeks.

#### 2.4.5 Mindful Attention Awareness Scale (MAAS)

A self-report questionnaire to value the mindfulness construct of “presence or absence of attention and awareness in the present moment” [27]. The MAAS consists of 15 items that can value the individual differences in frequency of mindful states and produce a total score of mindfulness. Participants have to indicate how frequently they have the experience shown in each item with a score from 1 to 6 on a Likert scale: 1 (almost always) to 6 (almost never). The answer to each item suggests how often the person feels he is performing the task in an automatic way without much awareness, how much he/she is concentrating on and in charge of what he/she is doing or conversely how much he/she is not paying attention to what is happening.

### 2.5 Optimized Voxel-Based Morphometry

For all preprocessing and analysis steps, the SPM software (Wellcome Department of Cognitive Neurology, University College London) and MATLAB 7.5 (MathWorks, MA, USA) were used. In order to obtain a better estimation of brain tissues maps, we implemented an optimized voxel-based morphometry (VBM) protocol for segmentation and normalization processes, using the DARTEL (Diffeomorphic Anatomical Registration using Exponentiated Lie algebra) toolbox for SPM [18]. Briefly, this approach is based on the creation of a customized anatomical template built directly from participants’ T1-weighted images instead of the canonical one provided with SPM (MNI template, ICBM 152, Montreal Neurological Institute). This allows us to obtain a finer normalization into standard space and consequently to avoid brain region volume under/over estimations, which may be induced by the adoption of an external template. Moreover, we applied a modified VBM analysis specifically designed for longitudinal studies, by using the VBM8 toolbox (http://dbm.neuro.uni-jena.de/) which allows to control for potential artifacts induced by the normalization process. Briefly, all the images of each participant are registered to correct for position (but not size) and the normalization estimates are derived from the first (baseline) scan only. The estimated normalization parameters are then applied to all images of one subject.

The hidden Markov Random Field model was applied in all segmentation processes in order to remove isolated voxels. Grey matter maps have been spatially normalized using a modulation procedure, which leads to an estimation of the absolute volume of grey matter structures (gray matter volume - GMV). After spatial normalization, the data were smoothed with an 8 mm FWHM (full width at half maximum) Gaussian kernel.

### 2.6 Voxel-Based Cortical Thickness

The VBCT toolbox for SPM was used to calculate grey matter cortical thickness. Here the thickness is calculated using segmented MR images in the subject’s native space, assigning an absolute measure of cortical thickness to each grey matter voxel. Differently from surface-based methods, voxel-based cortical thickness measurements do not require the construction of a three-dimensional surface model. Grey and white matter boundaries are instead defined on the basis of voxel information [19], [28] and cortical thickness is then calculated at every volumetric point within the cortex (defined as 0.5 probability of being GM), based on the length of the trajectory from inner to outer boundaries. To obtain an accurate spatial normalization, deformation fields obtained from normalization of GM probability maps to the average size template and then to MNI were also applied to obtained VBCT maps. A surface-based 8 mm FWHM smoothing was consequently applied to VBCT maps. Regionally-specific differences in cortical thickness between pre and post MBSR maps were then compared on a voxel-by-voxel basis.

### 2.7 Statistical analyses of imaging data

Three repeated measures analysis of covariance (RP ANCOVA) models were performed using the Statistical Package for the Social Sciences (SPSS 19.0), testing for differences in (1) GMV, and (3) VBCT values, using “Group” (MBSR and wait-list controls) as the between-subjects factor and “Pre-Post” (MRI PRE and POST) as the within-subjects factor. Age, gender and total brain volume (TBV) (i.e. the sum of gray and white tissue maps) were included as covariates in all analyses. Multiple comparisons correction was performed using a Montecarlo simulation (corrected p<.05) based on the probability of false-positive detection which takes into account both the individual voxel probability threshold and voxel cluster size (cluster connection radius = 4 mm, individual voxel threshold p<.001, iterations = 1000, FWHM = 8 mm, inclusive masks obtained by averaging participants baseline grey matter tissue maps). Moreover, to investigate possible interactions between psychological dimensions and brain morphometric changes in response to MBSR, a correlational analysis between psychological scores and statistically significant GMV/VBCT clusters was performed. Individual average thickness or volume values were extracted from each significant anatomical cluster using the Rex toolbox for SPM (http://web.mit.edu/swg/software.htm) and Pearson product/moment “r” coefficients were calculated using three different combinations (Bonferroni correction for multiple comparisons, p<.05): pre-MBSR (i.e. T0) brain volumes and pre-post MBSR differences (i.e. Δ) in psychological scores (T0-Brain/ΔPsy); pre-post MBSR differences in both brain volumes and psychological scores (ΔBrain/ΔPsy); pre-post MBSR differences in brain volumes and psychological scores before MBSR training (ΔBrain/T0-Psy). Specifically, we tried to unveil possible interactions between the effect of MBSR training on (i) both psychological and neuroanatomical profiles of each subject (pre-post interaction), or (ii) their pre-MBSR psychological profile (testing for possible predispositions to MBSR training).

### 2.8 Multivariate nodal thickness classification

In order to obtain a global overview of the impact of the MBSR program over cortical structures we also performed a multivariate pattern analysis classification procedure on the cortical thickness data. Trying to discriminate MBSR trainees and wait-list subjects on the basis of their anatomical changes across the 8 weeks, we calculated the difference between pre-MBSR and post-MBSR whole brain cortical thickness values for each participant. Whole brain map has been consequently parcellated into 90 regions according to the AAL atlas [29], and regional thickness differences were used as classification parameters (features of brain response to MBSR). Using Weka software [30]–[32], a support vector machine (SVM) algorithm was tested through leave-one-out cross-validation, resulting in an estimation of the overall correct classification percentage (% Accuracy into discriminate subjects who attended the course; Sensitivity; Specificity) as well as in a regional specific discriminative weight, with the expression of each region contributing to the overall classification process.

## Results

One participant from the MBSR group and two participants from the control group did not attend the second MRI acquisition, so the MBSR and control groups entered into the analyses were composed respectively by 23 (12F/11M) and 22 (11F/11M) subjects. Results of the between-groups comparison of age, gender and total brain volume are reported in Table 1.

**Table 1:** Groups demographics and psychological changes after MBSR.

	MBSR				Wait-list			*p*
	T0	T1	F	*p*	T0	T1	F	
	(M±SD)				(M±SD)			
**Age**	31±4	–	–	–	30±4	–	–	–
**Gender**	12 F, 11 M	–	–	–	11 F, 11 M	–	–	–
**Total brain volume (cm^3^)**	1467±232	–	–	–	1398±255	–	–	–
**MAAS**	62±16	65±10	-.534	.597	63±6	62±9	.312	.558
**PSWQ**	43±10	35±8	2.665	**.** ***012***	41±1	40±2	.347	.260
**STAI_State**	38±9	32±6	2.259	**.** ***031***	37±2	36±6	.345	.220
**STAI_Trait**	40±9	38±8	.716	.479	39±2	39±4	.233	.370
**TAS**	48±14	34±12	3.142	**.** ***004***	45±3	44±6	.413	.450
**BDI**	9±5	5±6	2.086	**.** ***046***	7±4	7±2	.134	.480

Table 1 shows demographic data and pre (t0) – post (t1) MBSR changes in psychological dimensions of both groups of participants. P-values refer to a repeated measure ANCOVA (p<.05).

### 3.1 Psychological evaluation

Results of the psychometric test comparisons are displayed in Table 1. Briefly, participants in the two groups did not differ for age, gender distribution or TBV. MBSR trainees showed a significant pre-post decrease in alexithymia (t_(22)_ = 3.142; p<.004), worry (t_(22)_ = 2.665; p<.012), state anxiety (t_(22)_ = 2.259; p<.031) and depression levels (t_(22)_ = 2.086; p<.046), while no differences were detected by the MAAS (t_(22)_ = 0.879; p<.418). No differences were found for participants in the control group (p<.05 corrected).

### 3.2 Morphovolumetric Results

#### 3.2.1 VBCT and VBM comparisons

**Figure 1:**
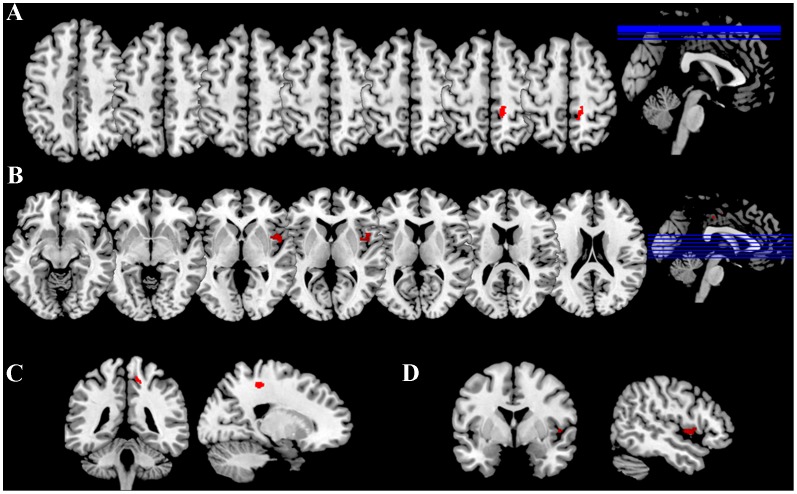
Morphometric changes after MBSR. Panels A, B, C and D show the results of voxel-wise cortical thickness comparison between MBSR trainees and control-group participants (repeated measures ANCOVA, p<.05, Montecarlo correction for multiple comparisons; individual VBCT masks), with clusters of increased thickness plotted on a reference T1-weighted image in MNI space and radiological convention. Specifically, panels A and C show axial, coronal and sagittal views of right somatosensory cluster, while B and D show the cluster located in the right insula.

**Figure 2:**
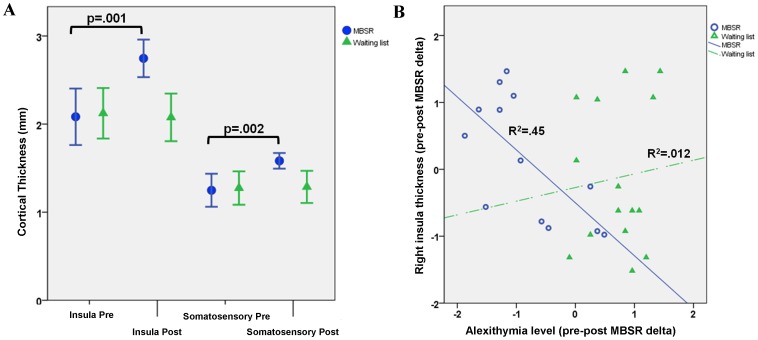
Pre-Post MBSR thickness comparisons and correlation between psychological and morphometric results. Panel A reports insula and somatosensory cortex average cortical thickness values for both groups, calculated before and after the MBSR/wait-list periods. Significant pre-post differences for MBSR group participants obtained using a repeated measures ANOVA model are reported. Scatterplot in panel B refers to the significant inverse correlation between the pre-post MBSR change in insula cortical thickness (y axis) and participants alexithymia level (x axis) (ΔBrain/ΔPsy index, z-scores, MBSR group R^2^ = .45, wait-list group R^2^ = .012).

A repeated measures ANCOVA (rpAN COVA) was performed on GMV and VBCT values (two groups×two time-points), with age, gender and TBV as covariates. The group×time interaction was significant for the VBCT model (F _(1,25)_ = 5.456; p = .008; η^2^ = .467), indicating an increases in cortical thickness greater in the MBSR than in the control group, specifically located in the right insula (F _(1,25)_ = 12.134; p* = *.001) and somatosensory cortex (F _(1,25)_ = 10.316; p = .002) (Figure 1, Figure 2A, Table 2). Change in cortical thickness in the control group were not significant for either insula (F _(1,25)_ = −1.12; p = .44) and somatosensory cortex (F _(1,25)_ = 0.78; p = .65). No clusters of GMV reached the statistical significance threshold either for main or interaction effects.

**Table 2:** Optimized morphovolumetric analysis results.

Increased cortical thickness	*voxels*	MNI coordinates			Peak F-value
		*x*	*y*	*z*	
***Cluster 1***	**178**				
Right somatosensory cortex		3	−22	46	3.14
Right Paracentral Lobule		9	−40	61	3.01
***Cluster 2***	**133**				
Right Insula Lobe		45	6	1	3.35
Right Inferior Frontal Gyrus (p. Opercularis)		48	11	4	3.23

Table 2 reports anatomical clusters of increased cortical thickness values after MBSR exposure. Voxel count, MNI coordinates and peak F-values are reported. Results referred to paired t-statistics obtained using a multiple comparisons correction based on a Montecarlo simulation with a corrected p<.05.

#### 3.2.2 Correlation between morphovolumetric and psychological data

The Rex toolbox for SPM was used to extract individual cortical thickness values from the insula and somatosensory clusters obtained in the rpANCOVA model. The rate of changes in thickness were then correlated with the changes in psychological indexes of interest (ΔBrain/ΔPsy), revealing a significant negative correlation for alexithymia level and insula cluster thickness values (*r* = −0.712; p<.01) in MBSR subjects after the training (Figure 2B). To verify that such correlation was not due to spurious variability in brain volume estimations, the same analysis was repeated in control subjects with no significant results. Moreover, it is noteworthy that correlational analyses between multiple, longitudinal measurements might induce statistical artefact due to the “regression to the mean” (RTM) phenomenon [33]. Briefly, this occurs when multiple acquisitions are made on the same subject, leading to the inherent tendency to obtain measurements fluctuating around the population’s mean (such as in the repeated measurement paradigm we applied), with an high probability of detecting values close to the mean if extreme values have been detected during the first measurement (and viceversa). Thus, in order to control for such statistical artifact, additional control analyses were performed by calculating the within-subjects variance of psychological and anatomical measurements, and quantifying the RTM effect using the approach suggested in [33]. No significant changes in the correlation coefficients were detected after compensating for RTM influence.

#### 3.2.4 Identification of the most discriminative brain regions

**Figure 3:**
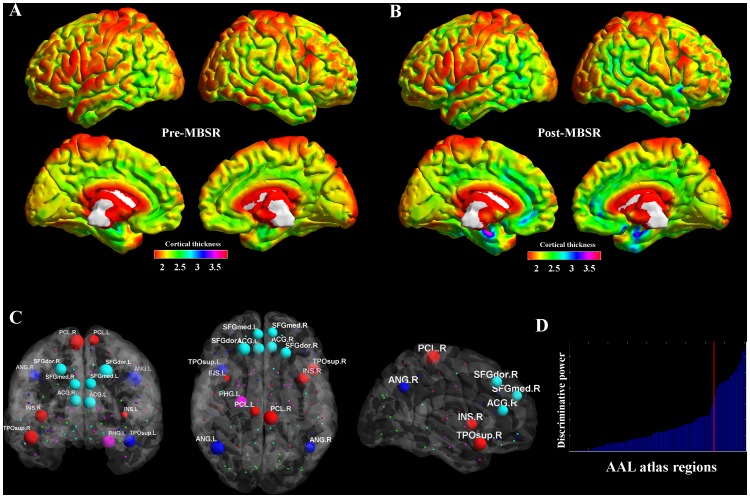
Cortical thickness based multivariate pattern classification. Panel A and B report a surface-based representation of average cortical thickness values for MBSR-trainees before and after the course. By computing the pre-post MBSR difference in cortical thickness level for both MBSR trainees and wait-list subjects, and by calculating this value for each of the 90 regions composing the AAL anatomical atlas, a support vector machine algorithm has been trained into discriminate the two groups. Results indicates an average identification accuracy equal to 83.32% (CI = .6346–.89; accuracy = .79; Sensitivity = .72; Specificity = .89), with panel C and D respectively report coronal, axial and sagittal views of a glass brain rendering showing the most discriminative regions (n = 15) and the distribution of discriminative power across regions (red line = 90th° percentile). Abbreviations: ANG = Angular gyrus; PCL = Paracentral lobule; SFGmed = Superior frontal gyrus medial part; SFGdor = Superior frontal gyrus dorsal part; ACG = Anterior cingulate gyrus; INS = Insula lobe; PHG = Parahippocampal gyrus; TPOsup = superior Temporal pole.

SVM classification indicated a pattern of brain regions that discriminated MBSR trainees from control subjects with a correct classification rate of 83.32% (confidence interval [CI]:.6346–.89; accuracy = .79; Sensitivity = .72; Specificity = .89). Features that overcame the 90th percentile were plotted on a three-dimensional glass brain in order to show brain areas that contributed the most to subjects identification (Fig. 3C & D). The classifier weights were higher in a group of regions located in the bilateral anterior cingulate cortex, bilateral insula, bilateral superior frontal gyrus (medial part), bilateral middle frontal gyrus, bilateral temporal pole, bilateral angular gyrus, bilateral precentral gyrus, left parahippocampal gyrus. Results are plotted onto a glass cortical surface using the BrainNet Viewer toolbox for SPM (http://www.nitrc.org/projects/bnv/).

## Discussion

The last decade has revealed growing evidence of anatomical and functional brain modifications associated with meditation practices, with results referring to a large variety of different methods of meditation. Noteworthy, evidence is often derived from cross-sectional studies based on subjects with a long-history of meditation practice. Moreover, the link between the behavioral effect of meditation and the neuroanatomical/functional changes observed in neuroimaging studies has not being explored in details. Here, we longitudinally examined the effects of a brief 8-week MBSR training program [21] on both neuroanatomical and psychological variables of meditation naïve subjects. We found an increase of cortical thickness in the right insular lobe and somatosensory cortex of meditation-naïve participants after the MBSR training, as well a significant after-training reduction of several psychological indices related to worry, state anxiety, depression and alexithymia. Finally, a significant negative correlation between alexithymia level and pre-post MBSR changes in insular thickness was also observed.

Both brain imaging and electrophysiological studies have suggested that right insula is a key node for interoception, awareness of body movements and emotional awareness [34], due to its extensive viscera-sensory inputs from the periphery and reciprocal connections with limbic, somatosensory, prefrontal and temporal cortices [35], [36]. The insula has been specifically linked to the monitoring of visceral parts of the body, and its possible role into the re-representation of interoception offers a possible basis for its involvement in all subjective feelings [37]. Following the first cross-sectional evidence of increased right insula thickness in expert meditators (>1000 hours of meditation) observed by Lazar and colleagues (2005), other studies have shown a modulation of insula structural and functional properties in expert meditators. For instance, Hölzel et al. found a significantly greater grey matter density in right insula of Vipassana meditators compared to controls [12], while Grant and colleagues documented increased insula thickness in Zen meditators [15].

As for MBSR, using a fine anatomical parcellation analysis of insula activity, Farb and colleagues [38] showed higher activation of the anterior dysgranular insular regions in MBSR trainees with respect to controls during an interoceptive attention task, while in another study they demonstrated a reduced insula deactivation in MBSR practitioners during a sadness provocation task, which may be interpreted as increased interoceptive awareness and thus a lesser propensity to process highly-emotional incoming stimuli [39]. Moreover, in a recent study Murakami and colleagues (2012) also demonstrated a significant correlations between right anterior insula volume and individual scores on a self-report questionnaire investigating mindfulness-related dimensions like “non-reactivity to inner experience, non-judging, acting with awareness, describing, and observing” [40]. Accordingly, the increase of insula thickness observed in our participants may be the result of their increased awareness (through paying attention moment to moment in a non-judgmental way) of bodily sensations (from the most subtle to the most evident). Interestingly, the insular lobe seems to be also involved in the cognitive processing of nociceptive stimuli, with an increase in its activity (measured using arterial spin labeling after just 4 days of mindfulness training) associated with a significantly diminished pain perception in healthy subjects [41]. In conclusion, our results confirm insula high responsiveness to MBSR and put forward the feasibility of a plasticity-related response even after a brief mindfulness training exposure.

We also observed an MBSR-induced thickness change in right somatosensory cortex, a region associated with sensory-discriminative processing of nociceptive information [42]. As with the insula, this region is often associated with meditation practice, with findings of increased activation during experiential focusing [43] and observation of neutral and sad clips [39] in MBSR trainees. During the MBSR program, focusing attention on somatic sensations is trained through “body scanning“, sitting meditation, mindful yoga and walking meditation exercises, in which students learn to focus mindful attention on sensations coming from different parts of their bodies. Accordingly, as suggested by evidence of increased alpha-band modulation after mindfulness training observed in this region using magneto-electroencephalography (MEG) [44], repeated increases of average activation in the somatosensory cortex during the program could be responsible for the observed effect.

Multivariate pattern analysis revealed a large number of regions which seem to be highly informative for the identification of MBSR trainees. Interestingly, this pool of regions, encompassing frontal lobe, anterior cingulate cortex, insula, temporal pole, somatosensory cortex, angular and parahippocampal gyri, have been separately reported in previous anatomical or functional studies on different types of meditation [45], [46]. In light of the sensory and cognitive functions they are associated with, these regions may compose an unspecific network of areas that are highly sensitive to meditation practicing in general. Besides morphometric changes, a significant after-training reduction of several psychological indexes related to worry, state anxiety, depression and alexithymia were observed, while no significant changes or correlation with the MAAS were detected. Given the theoretically framework sustaining the items proposed in the MAAS, which seem to describe the capacity of the subject to pay attention in a very acute way, rather than to “hold in awareness- paying attention in a non-judgmental way- the universe of internal and external events as they unfold” as mindfulness theory suggests, this is not completely surprising (see Grossman [10]). More interestingly, the correlation between alexithymia levels and anatomical changes after mindfulness exposure gives reason of previous evidences correlating the insula and alexithymia levels in several morphometric studies not specifically involving meditation. For instance, in a positron emission tomography (PET) study, Kano and colleagues highlighted how individuals scoring higher than the clinical cut-off on the TAS-20 showed hyperactivity of the right insula [47], interpreting this hyperactivation as the tendency of these individuals to exacerbate physical illness, possibly caused by an unpleasant internal signal amplification. Moreover, in a study using film clips to elicit emotional responses [48], the authors documented increased activity in the bilateral insula in TAS-20 high-scorers compared to low-scorers, along with increased sensory and motor cortices activity. Indeed, the functions associated with the insula seems to resemble the mental state promoted through mindfulness, while they are basically opposite to the theoretical description of alexithymia, which is intended as a reduction or incapacity to experience or verbalize emotions [49]. A similar finding regarding the insula has been documented by Farb and colleagues [43], by looking at differences in brain activation in response to different types of self-reference monitoring tasks in both novices and MBSR-trained subjects. While a strong coupling between the right insula and self-referential cortical midline regions (medial prefrontal cortex - mPFC) has been observed in novices during experiential focus task, an increase in the activity of a right lateralized network comprising the lateral prefrontal cortex (PFC) and viscerosomatic areas such as the insula, secondary somatosensory cortex and inferior parietal lobule seems to characterize the response of MBSR-trained participants, also leading to the uncoupling of the aforementioned insula-mPFC connectivity. Such evidence suggests how the integration of visceral/emotional information in MBSR practitioners may be disrupted and dissociated by self-referencing activity, an effect which may plausibly explain the decrease in alexithymia level observed in our study.

The MBSR program, by training interoception, may improve the understanding and processing of own emotional reactions to internal and external stimuli, with a positive cascade effect on individual ability to exert cognitive control over emotions. Therefore, the insula-alexithymia interaction observed in the participants to the MBSR program could simply indicate an increase in their emotional awareness driven by the mindfulness experience [50], a phenomena that seems to be neuro-biologically supported by an increase in the activity of the insula [39], which could in turn plausibly lead, if expressed over time, to local neurogenesis. However, regarding the cellular underpinnings of the observed structural changes, there is no evidence of neuronal proliferation in the human cortex during most of post-natal development, thus making unlikely that cortical thickness changes observed after MBSR training are due to changes in the number of cortical neurons [51]. Plausibly such alterations rely on changes in the amount of glial and capillary support, as well as in dendritic arborization and cortical capillary density [52], [53]. Consistently, gliogenesis as a consequence of learning and experience has been already demonstrated [54] and candidate as a possible mechanism for experience-related changes in gray matter morphology [55]. Importantly, as any other intervention based on learning a new method and its practice, the identification of treatment specific properties and its behavioural/biological effects should be achieved via the comparison with an adequate control condition. Previous literature about MBSR training has included different solutions to address this issue, with the vast majority of the studies adopting a wait-control list approach [see 10,56–57]. Interestingly, recent evidences suggest how an active control condition might be more adequate to identify the active ingredients responsible for MBSR training efficacy into modulating, for instance, pain perception [58] and stress response [59]. Even though our design includes a wait-control group, thus requiring further studies in order to confirm the impact of MBSR program against an active control condition as the one suggested by MacCoon and colleagues (2012) [59], the anatomical localization of those regions showing a significant increase in thickness after the training as well as their extensively documented functional involvement during MBSR-like tasks using fMRI [43], [45], [46], denote our results as a proof of the efficacy of MBSR training both on participants’ psychological well-being and on cortical grey matter structures in naïve subjects. Moreover, the link with alexithymia levels also posits the existence of a specific neuroanatomical substrate linking mindfulness training and an increase in subjective awareness of their own emotions and feelings.

## References

[1] Kabat-Zinn J, Lipworth L, Burney R (1985) The clinical use of mindfulness meditation for the self-regulation of chronic pain. J Behav Med 8: 163–190. 3897551 10.1007/BF00845519

[2] Kabat-Zinn J, Massion AO, Kristeller J, Peterson LG, Fletcher KE, et al (1992) Effectiveness of a meditation-based stress reduction program in the treatment of anxiety disorders. Am J Psychiatry 149: 936–943. 1609875 10.1176/ajp.149.7.936

[3] Chiesa A, Serretti A (2009) Mindfulness-based stress reduction for stress management in healthy people: a review and meta-analysis. J Altern Complement Med 15: 593–600 10.1089/acm.2008.0495 [doi]. 19432513 10.1089/acm.2008.0495

[4] Irving JA, Dobkin PL, Park J (2009) Cultivating mindfulness in health care professionals: a review of empirical studies of mindfulness-based stress reduction (MBSR). Complement Ther Clin Pract 15: 61–66 S1744-3881(09)00005-X [pii];10.1016/j.ctcp.2009.01.002 [doi]. 19341981 10.1016/j.ctcp.2009.01.002

[5] Campbell TS, Labelle LE, Bacon SL, Faris P, Carlson LE (2012) Impact of Mindfulness-Based Stress Reduction (MBSR) on attention, rumination and resting blood pressure in women with cancer: a waitlist-controlled study. J Behav Med 35: 262–271 10.1007/s10865-011-9357-1 [doi]. 21667281 10.1007/s10865-011-9357-1

[6] Roemer L, Lee JK, Salters-Pedneault K, Erisman SM, Orsillo SM, et al (2009) Mindfulness and emotion regulation difficulties in generalized anxiety disorder: preliminary evidence for independent and overlapping contributions. Behav Ther 40: 142–154 S0005-7894(08)00065-8 [pii];10.1016/j.beth.2008.04.001 [doi]. 19433145 10.1016/j.beth.2008.04.001 PMC3719394

[7] Paul NA, Stanton SJ, Greeson JM, Smoski MJ, Wang L (2013) Psychological and neural mechanisms of trait mindfulness in reducing depression vulnerability. Soc Cogn Affect Neurosci 8: 56–64 nss070 [pii];10.1093/scan/nss070 [doi]. 22717383 10.1093/scan/nss070 PMC3541493

[8] Teasdale JD, Segal ZV, Williams JM, Ridgeway VA, Soulsby JM, et al (2000) Prevention of relapse/recurrence in major depression by mindfulness-based cognitive therapy. J Consult Clin Psychol 68: 615–623. 10965637 10.1037//0022-006x.68.4.615

[9] Bowen S, Witkiewitz K, Dillworth TM, Chawla N, Simpson TL, et al (2006) Mindfulness meditation and substance use in an incarcerated population. Psychol Addict Behav 20: 343–347 2006-10832-015 [pii];10.1037/0893-164X.20.3.343 [doi]. 16938074 10.1037/0893-164X.20.3.343

[10] Grossman P, Tiefenthaler-Gilmer U, Raysz A, Kesper U (2007) Mindfulness training as an intervention for fibromyalgia: evidence of postintervention and 3-year follow-up benefits in well-being. Psychother Psychosom 76: 226–233 000101501 [pii];10.1159/000101501 [doi]. 17570961 10.1159/000101501

[11] Lazar SW, Kerr CE, Wasserman RH, Gray JR, Greve DN, et al.. (2005) Meditation experience is associated with increased cortical thickness. Neuroreport 16: 1893–1897. 00001756-200511280-00005 [pii]. 10.1097/01.wnr.0000186598.66243.19 PMC1361002 16272874

[12] Hölzel BK, Ott U, Gard T, Hempel H, Weygandt M, et al (2008) Investigation of mindfulness meditation practitioners with voxel-based morphometry. Soc Cogn Affect Neurosci 3: 55–61 nsm038 [pii];10.1093/scan/nsm038 [doi]. 19015095 10.1093/scan/nsm038 PMC2569815

[13] Vestergaard-Poulsen P, van BM, Skewes J, Bjarkam CR, Stubberup M, et al (2009) Long-term meditation is associated with increased gray matter density in the brain stem. Neuroreport 20: 170–174 10.1097/WNR.0b013e328320012a [doi]. 19104459 10.1097/WNR.0b013e328320012a

[14] Luders E, Toga AW, Lepore N, Gaser C (2009) The underlying anatomical correlates of long-term meditation: larger hippocampal and frontal volumes of gray matter. Neuroimage 45: 672–678. 19280691 10.1016/j.neuroimage.2008.12.061 PMC3184843

[15] Grant JA, Courtemanche J, Duerden EG, Duncan GH, Rainville P (2010) Cortical thickness and pain sensitivity in zen meditators. Emotion 10: 43–53 2010-01983-010 [pii];10.1037/a0018334 [doi]. 20141301 10.1037/a0018334

[16] Hölzel BK, Carmody J, Vangel M, Congleton C, Yerramsetti SM, et al (2011) Mindfulness practice leads to increases in regional brain gray matter density. Psychiatry Res 191: 36–43 S0925-4927(10)00288-X [pii];10.1016/j.pscychresns.2010.08.006 [doi]. 21071182 10.1016/j.pscychresns.2010.08.006 PMC3004979

[17] Wright IC, McGuire PK, Poline JB, Travere JM, Murray RM, et al (1995) A voxel-based method for the statistical analysis of gray and white matter density applied to schizophrenia. Neuroimage 2: 244–252 S1053-8119(85)71032-4 [pii];10.1006/nimg.1995.1032 [doi]. 9343609 10.1006/nimg.1995.1032

[18] Ashburner J (2007) A fast diffeomorphic image registration algorithm. Neuroimage 38: 95–113 S1053-8119(07)00584-8 [pii];10.1016/j.neuroimage.2007.07.007 [doi]. 17761438 10.1016/j.neuroimage.2007.07.007

[19] Hutton C, De VE, Ashburner J, Deichmann R, Turner R (2008) Voxel-based cortical thickness measurements in MRI. Neuroimage 40: 1701–1710 S1053-8119(08)00080-3 [pii];10.1016/j.neuroimage.2008.01.027 [doi]. 18325790 10.1016/j.neuroimage.2008.01.027 PMC2330066

[20] Hutton C, Draganski B, Ashburner J, Weiskopf N (2009) A comparison between voxel-based cortical thickness and voxel-based morphometry in normal aging. Neuroimage 48: 371–380 S1053-8119(09)00679-X [pii];10.1016/j.neuroimage.2009.06.043 [doi]. 19559801 10.1016/j.neuroimage.2009.06.043 PMC2741580

[21] Kabat-Zinn J (1982) An outpatient program in behavioral medicine for chronic pain patients based on the practice of mindfulness meditation: theoretical considerations and preliminary results. Gen Hosp Psychiatry 4: 33–47. 7042457 10.1016/0163-8343(82)90026-3

[22] Bagby RM, Parker JD, Taylor GJ (1994) The twenty-item Toronto Alexithymia Scale-I. Item selection and cross-validation of the factor structure. J Psychosom Res 38: 23–32. 8126686 10.1016/0022-3999(94)90005-1

[23] Bressi C, Taylor G, Parker J, Bressi S, Brambilla V, et al.. (1996) Cross validation of the factor structure of the 20-item Toronto Alexithymia Scale: an Italian multicenter study. J Psychosom Res 41: 551–559. S0022399996002280 [pii]. 10.1016/s0022-3999(96)00228-0 9032718

[24] Meyer TJ, Miller ML, Metzger RL, Borkovec TD (1990) Development and validation of the Penn State Worry Questionnaire. Behav Res Ther 28: 487–495. 0005-7967(90)90135-6 [pii]. 10.1016/0005-7967(90)90135-6 2076086

[25] Vigneau F, Cormier S (2008) The factor structure of the State-Trait Anxiety Inventory: an alternative view. J Pers Assess 90: 280–285 792276383 [pii];10.1080/00223890701885027 [doi]. 18444124 10.1080/00223890701885027

[26] Beck AT, Steer RA, Ball R, Ranieri W (1996) Comparison of Beck Depression Inventories -IA and -II in psychiatric outpatients. J Pers Assess 67: 588–597 10.1207/s15327752jpa6703_13 [doi]. 8991972 10.1207/s15327752jpa6703_13

[27] Brown KW, Ryan RM (2003) The benefits of being present: mindfulness and its role in psychological well-being. J Pers Soc Psychol 84: 822–848. 12703651 10.1037/0022-3514.84.4.822

[28] Yezzi AJ Jr, Prince JL (2003) An Eulerian PDE approach for computing tissue thickness. IEEE Trans Med Imaging 22: 1332–1339 10.1109/TMI.2003.817775 [doi]. 14552586 10.1109/TMI.2003.817775

[29] Tzourio-Mazoyer N, Landeau B, Papathanassiou D, Crivello F, Etard O, et al (2002) Automated anatomical labeling of activations in SPM using a macroscopic anatomical parcellation of the MNI MRI single-subject brain. Neuroimage 15: 273–289 10.1006/nimg.2001.0978 [doi];S1053811901909784 [pii]. 11771995 10.1006/nimg.2001.0978

[30] Frank E, Hall M, Trigg L, Holmes G, Witten IH (2004) Data mining in bioinformatics using Weka. Bioinformatics 20: 2479–2481 10.1093/bioinformatics/bth261 [doi];bth261 [pii]. 15073010 10.1093/bioinformatics/bth261

[31] Rubinov M, Sporns O (2010) Complex network measures of brain connectivity: uses and interpretations. Neuroimage 52: 1059–1069 S1053-8119(09)01074-X [pii];10.1016/j.neuroimage.2009.10.003 [doi]. 19819337 10.1016/j.neuroimage.2009.10.003

[32] Watts DJ, Strogatz SH (1998) Collective dynamics of ‘small-world' networks. Nature 393: 440–442 10.1038/30918 [doi]. 9623998 10.1038/30918

[33] Barnett AG, van der Pols JC, Dobson AJ (2005) Regression to the mean: what it is and how to deal with it. Int J Epidemiol 34: 215–220 10.1093/ije/dyh299 [doi];dyh299 [pii]. 15333621 10.1093/ije/dyh299

[34] Craig AD (2009) How do you feel–now? The anterior insula and human awareness. Nat Rev Neurosci 10: 59–70 nrn2555 [pii];10.1038/nrn2555 [doi]. 19096369 10.1038/nrn2555

[35] Augustine JR (1996) Circuitry and functional aspects of the insular lobe in primates including humans. Brain Res Brain Res Rev 22: 229–244. S0165017396000112 [pii]. 10.1016/s0165-0173(96)00011-2 8957561

[36] Mesulam MM, Mufson EJ (1982) Insula of the old world monkey. III: Efferent cortical output and comments on function. J Comp Neurol 212: 38–52 10.1002/cne.902120104 [doi]. 7174907 10.1002/cne.902120104

[37] Nieuwenhuys R (2012) The insular cortex: a review. Prog Brain Res 195: 123–163 B978-0-444-53860-4.00007-6 [pii];10.1016/B978-0-444-53860-4.00007-6 [doi]. 22230626 10.1016/B978-0-444-53860-4.00007-6

[38] Farb NA, Segal ZV, Anderson AK (2013) Mindfulness meditation training alters cortical representations of interoceptive attention. Soc Cogn Affect Neurosci 8: 15–26 nss066 [pii];10.1093/scan/nss066 [doi]. 22689216 10.1093/scan/nss066 PMC3541492

[39] Farb NA, Anderson AK, Mayberg H, Bean J, McKeon D, et al (2010) Minding one's emotions: mindfulness training alters the neural expression of sadness. Emotion 10: 25–33 2010-01983-008 [pii];10.1037/a0017151 [doi]. 20141299 10.1037/a0017151 PMC5017873

[40] Murakami H, Nakao T, Matsunaga M, Kasuya Y, Shinoda J, et al (2012) The structure of mindful brain. PLoS One 7: e46377 10.1371/journal.pone.0046377 [doi];PONE-D-12-21884 [pii]. 23029500 10.1371/journal.pone.0046377 PMC3460809

[41] Zeidan F, Martucci KT, Kraft RA, Gordon NS, McHaffie JG, et al (2011) Brain mechanisms supporting the modulation of pain by mindfulness meditation. J Neurosci 31: 5540–5548 31/14/5540 [pii];10.1523/JNEUROSCI.5791-10.2011 [doi]. 21471390 10.1523/JNEUROSCI.5791-10.2011 PMC3090218

[42] Coghill RC, Sang CN, Maisog JM, Iadarola MJ (1999) Pain intensity processing within the human brain: a bilateral, distributed mechanism. J Neurophysiol 82: 1934–1943. 10515983 10.1152/jn.1999.82.4.1934

[43] Farb NA, Segal ZV, Mayberg H, Bean J, McKeon D, et al (2007) Attending to the present: mindfulness meditation reveals distinct neural modes of self-reference. Soc Cogn Affect Neurosci 2: 313–322 10.1093/scan/nsm030 [doi]. 18985137 10.1093/scan/nsm030 PMC2566754

[44] Kerr CE, Jones SR, Wan Q, Pritchett DL, Wasserman RH, et al (2011) Effects of mindfulness meditation training on anticipatory alpha modulation in primary somatosensory cortex. Brain Res Bull 85: 96–103 S0361-9230(11)00134-1 [pii];10.1016/j.brainresbull.2011.03.026 [doi]. 21501665 10.1016/j.brainresbull.2011.03.026

[45] Kang DH, Jo HJ, Jung WH, Kim SH, Jung YH, et al (2013) The effect of meditation on brain structure: cortical thickness mapping and diffusion tensor imaging. Soc Cogn Affect Neurosci 8: 27–33 nss056 [pii];10.1093/scan/nss056 [doi]. 22569185 10.1093/scan/nss056 PMC3541490

[46] Leung MK, Chan CC, Yin J, Lee CF, So KF, et al (2013) Increased gray matter volume in the right angular and posterior parahippocampal gyri in loving-kindness meditators. Soc Cogn Affect Neurosci 8: 34–39 nss076 [pii];10.1093/scan/nss076 [doi]. 22814662 10.1093/scan/nss076 PMC3541494

[47] Kano M, Hamaguchi T, Itoh M, Yanai K, Fukudo S (2007) Correlation between alexithymia and hypersensitivity to visceral stimulation in human. Pain 132: 252–263 S0304-3959(07)00064-4 [pii];10.1016/j.pain.2007.01.032 [doi]. 17360119 10.1016/j.pain.2007.01.032

[48] Karlsson H, Naatanen P, Stenman H (2008) Cortical activation in alexithymia as a response to emotional stimuli. Br J Psychiatry 192: 32–38 192/1/32 [pii];10.1192/bjp.bp.106.034728 [doi]. 18174507 10.1192/bjp.bp.106.034728

[49] Shipko S (1982) Alexithymia and somatization. Psychother Psychosom 37: 193–201. 7156304 10.1159/000287573

[50] Goldin PR, Gross JJ (2010) Effects of mindfulness-based stress reduction (MBSR) on emotion regulation in social anxiety disorder. Emotion 10: 83–91 2010-01983-016 [pii];10.1037/a0018441 [doi]. 20141305 10.1037/a0018441 PMC4203918

[51] Zatorre RJ, Fields RD, Johansen-Berg H (2012) Plasticity in gray and white: neuroimaging changes in brain structure during learning. Nat Neurosci 15: 528–536 nn.3045 [pii];10.1038/nn.3045 [doi]. 22426254 10.1038/nn.3045 PMC3660656

[52] Chklovskii DB, Mel BW, Svoboda K (2004) Cortical rewiring and information storage. Nature 431: 782–788 nature03012 [pii];10.1038/nature03012 [doi]. 15483599 10.1038/nature03012

[53] Thompson PM, Hayashi KM, Dutton RA, Chiang MC, Leow AD, et al (2007) Tracking Alzheimer's disease. Ann N Y Acad Sci 1097: 183–214 1097/1/183 [pii];10.1196/annals.1379.017 [doi]. 17413023 10.1196/annals.1379.017 PMC3197831

[54] Dong WK, Greenough WT (2004) Plasticity of nonneuronal brain tissue: roles in developmental disorders. Ment Retard Dev Disabil Res Rev 10: 85–90 10.1002/mrdd.20016 [doi]. 15362161 10.1002/mrdd.20016

[55] Zatorre RJ, Fields RD, Johansen-Berg H (2012) Plasticity in gray and white: neuroimaging changes in brain structure during learning. Nat Neurosci 15: 528–536 nn.3045 [pii];10.1038/nn.3045 [doi]. 22426254 10.1038/nn.3045 PMC3660656

[56] Kabat-Zinn J, Wheeler E, Light T, Skillings A, Scharf MJ, et al (1998) Influence of a mindfulness meditation-based stress reduction intervention on rates of skin clearing in patients with moderate to severe psoriasis undergoing phototherapy (UVB) and photochemotherapy (PUVA). Psychosom Med 60: 625–632. 9773769 10.1097/00006842-199809000-00020

[57] Davidson RJ, Kabat-Zinn J, Schumacher J, Rosenkranz M, Muller D, et al (2003) Alterations in brain and immune function produced by mindfulness meditation. Psychosom Med 65: 564–570. 12883106 10.1097/01.psy.0000077505.67574.e3

[58] Rosenkranz MA, Davidson RJ, Maccoon DG, Sheridan JF, Kalin NH, et al (2013) A comparison of mindfulness-based stress reduction and an active control in modulation of neurogenic inflammation. Brain Behav Immun 27: 174–184 S0889-1591(12)00475-8 [pii];10.1016/j.bbi.2012.10.013 [doi]. 23092711 10.1016/j.bbi.2012.10.013 PMC3518553

[59] MacCoon DG, Imel ZE, Rosenkranz MA, Sheftel JG, Weng HY, et al (2012) The validation of an active control intervention for Mindfulness Based Stress Reduction (MBSR). Behav Res Ther 50: 3–12 S0005-7967(11)00247-6 [pii];10.1016/j.brat.2011.10.011 [doi]. 22137364 10.1016/j.brat.2011.10.011 PMC3257026

