# Correction: Interaction between Neuroanatomical and Psychological Changes after Mindfulness-Based Training

**DOI:** 10.1371/journal.pone.0129754

**Published:** 2015-06-01

**Authors:** Emiliano Santarnecchi, Sicilia D’Arista, Eutizio Egiziano, Concetta Gardi, Roberta Petrosino, Giampaolo Vatti, Mario Reda, Alessandro Rossi

In the Author Contributions section, Eutizio Egiziano (EE) should be listed as one of the persons who wrote the paper.

## References

[1] Santarnecchi E, D’Arista S, Egiziano E, Gardi C, Petrosino R, Vatti G, et al (2014) Interaction between Neuroanatomical and Psychological Changes after Mindfulness-Based Training. PLoS ONE 9(10): e108359 doi: 10.1371/journal.pone.0108359 25330321 10.1371/journal.pone.0108359 PMC4203679

